# Characteristics of the Energetic Micro-initiator Through Integrating Al/Ni Nano-multilayers with Cu Film Bridge

**DOI:** 10.1186/s11671-016-1812-z

**Published:** 2017-01-13

**Authors:** Yuxin Zhang, Hongchuan Jiang, Xiaohui Zhao, Yichao Yan, Wanli Zhang, Yanrong Li

**Affiliations:** State Key Laboratory of Electronic Thin Films and Integrated Devices, University of Electronic Science and Technology of China, Chengdu, 610054 China

**Keywords:** Al/Ni multilayers, Micro-initiator, Nano-energetic, Electric explosion

## Abstract

An energetic micro-initiator through integrating Al/Ni nano-multilayers with Cu film bridge was investigated in this study. The Cu film bridge was initially fabricated with wet etching, and Al/Ni nano-multilayers were alternately deposited on the surface of Cu film bridge by magnetron sputtering. The periodic layer structure of Al/Ni nano-multilayers was verified by scanning electron microscopy. The exothermic reaction in Al/Ni nano-multilayers can be initiated with onset reaction temperature as low as 503 K, and the total reaction heat is about 774.6 J/g. This energetic micro-initiator exhibited improved performances with lower threshold voltage, smaller initiation energy, and higher explosion temperature compared with Cu film bridge. An extra violent explosion phenomenon with longer duration time and larger quantities of ejected product particles was detected on this energetic micro-initiator by high-speed camera. Overall, the electric explosion performances of Cu film bridge can be improved evidently with the integration of Al/Ni nano-multilayers.

## Background

Metal-based nano-energetic materials have attracted a lot of interests in recent years for their superior performances in terms of fast energy release rate, large amount of reaction heat, and more elements to choose [1–7]. Different nano-energetic materials based on intermetallic reaction or thermic reaction were used in various applications including initiation of secondary reactions [8], micro-initiator [9], welding and soldering [10], and airbags [11]. Many methods such as powder mixing, periodically deposition of multilayers, sol-gel, and arrested reactive milling have been introduced to fabricate nano-energetic materials [12]. Among these methods, periodic deposition of intermetallic multilayers provides a fascinating structure by integrating the energetic layers with microelectronic and mechanical systems (MEMS) to improve the performances with compact size, and the performances can be tuned easily by changing the number of layers and bilayer thickness period.

The requirements of electric ignition devices with miniaturization, low-energy initiation, and high performance have increased significantly in recent years; many efforts have been devoted on integrating nano-energetic materials with a film bridge initiator to improve ignition process and enhance energy output [13–18]. Currently, Al/Ni nano-multilayers are widely regarded as a type of promising nano-energetic material to integrate with MEMS for extremely high heating rates (10^5^–10^6^ K/S), fast combustion propagation velocities up to 10 m/s, and low onset reaction temperature (400–500 K) [19–22].

In this work, Al/Ni nano-multilayers were integrated with Cu film bridge to form an energetic micro-initiator. The structure and thermal properties of Al/Ni nano-multilayers were characterized by scanning electron microscopy (SEM), different scanning calorimetry (DSC), and X-ray diffraction (XRD). The effects of the presence of Al/Ni nano-multilayers on the electric explosion performances were systematically investigated.

## Methods

**Fig. 1:**
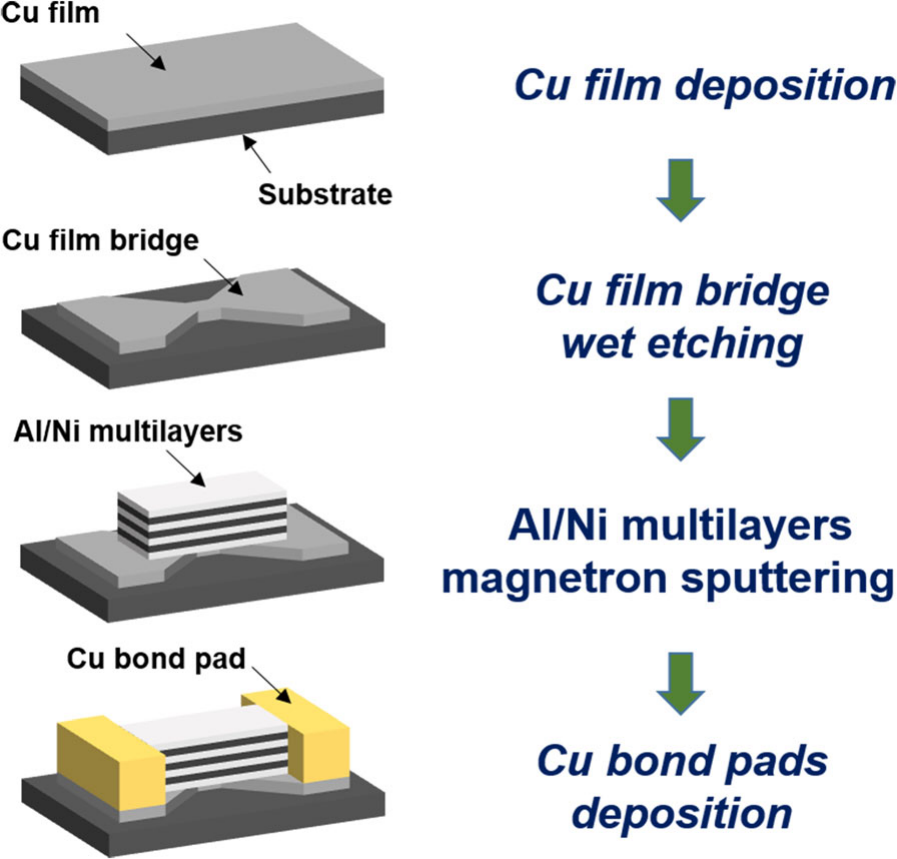
Fabrication process flow of Cu/Al/Ni-integrated film bridge

The fabrication process flow of Cu/Al/Ni-integrated film bridge is shown in Fig. 1. An alumina plate (0.5 mm thick) was used as the substrate, and first, it was ultrasonic-cleaned sequentially by using acetone, alcohol, and deionized water for 10 min. Then, the cleaned substrate was blow-dried by nitrogen gas and treated at 383 K for 40 min for further drying.

A pure Cu target foil (99.995 wt%) with the size of 100 mm was applied as target for sputtering. 2-μm-thick Cu layer was deposited on the alumina substrate with sputtering temperature, pressure, and power of 373 K, 0.55 Pa, and 100 W, respectively. Afterwards, positive photoresist was spin-coated onto the as-deposited Cu film and patterned with photolithography through a designed mask. Subsequently, FeCl_3_ solution was used to wet etching the exposed Cu film at room temperature. The dimension of the substrate is 10 mm (length) by 5 mm (width) by 0.5 mm (thickness). The dimension of the Cu film bridge is 0.6 mm (length) by 0.6 mm (width) by 2 μm (thickness).

After that, reversal photoresist was spin-coated onto the Cu film bridge, which was patterned using photolithography technology. The photoresist was exposed twice to generate a reentrant profile. Then, 2-μm-thick Al/Ni nano-multilayers with bilayer thickness of 200 nm (Al, 120 nm; Ni, 80 nm) were alternately deposited on the Cu film bridge by magnetron sputtering. The deposition parameters for Al layer and Ni layer were both set at 303 K, 0.4 Pa, and 100 W. The total thickness of Al/Ni nano-multilayers was determined by the number of layers. After removing the developed photoresist, two Cu bound pads were stacked on both sides of the Al/Ni nano-multilayers and lead wires were soldered for the connection to the voltage source.

The cross-sectional morphology of the Al/Ni nano-multilayers was characterized by SEM. The properties of Al/Ni nano-multilayers on the heat energy generation were measured by DSC, and the tests were carried out at a temperature range from 323 to 973 K with the heating rate of 10 K/min in flowing nitrogen. The phase information of the Al/Ni nano-multilayers before and after DSC experiments was determined by XRD.

**Fig. 2:**
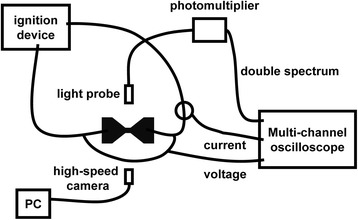
Schematic diagram of electric ignition measurement system

The electric explosion properties of the samples were tested by an electric ignition measurement system, as shown in Fig. 2. The discharge capacitor (0.22 μF) was used to apply voltage crossing micro-initiator. The temperature characteristics were determined by an electric explosion temperature diagnosis mode based on the “double-line atomic emission spectroscopy of a copper element” [23, 24]. The electric explosion performance and the reaction dynamic processes were recorded by high-speed camera with 20,000 frames per second.

## Results and Discussion

**Fig. 3:**
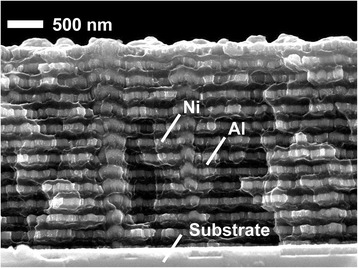
The cross-sectional image of the Al/Ni nano-multilayers with bilayer thickness of 200 nm

Figure 3 shows the cross-sectional image of the Al/Ni nano-multilayers with bilayer thickness of 200 nm (Al, 120 nm; Ni, 80 nm). We can see the well-aligned and periodic layer structure of Al layers and Ni layers. The planar layers remain continuous and homogenous, which are beneficial for the intermetallic reaction between Al layers and Ni layers to release energy.

**Fig. 4:**
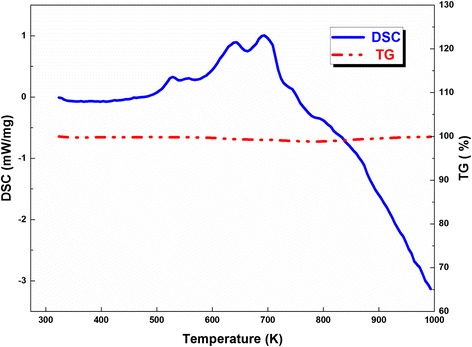
TG/DSC curves of Al/Ni nano-multilayers at the heating rate of 10 K/min in flowing nitrogen

**Fig. 5:**
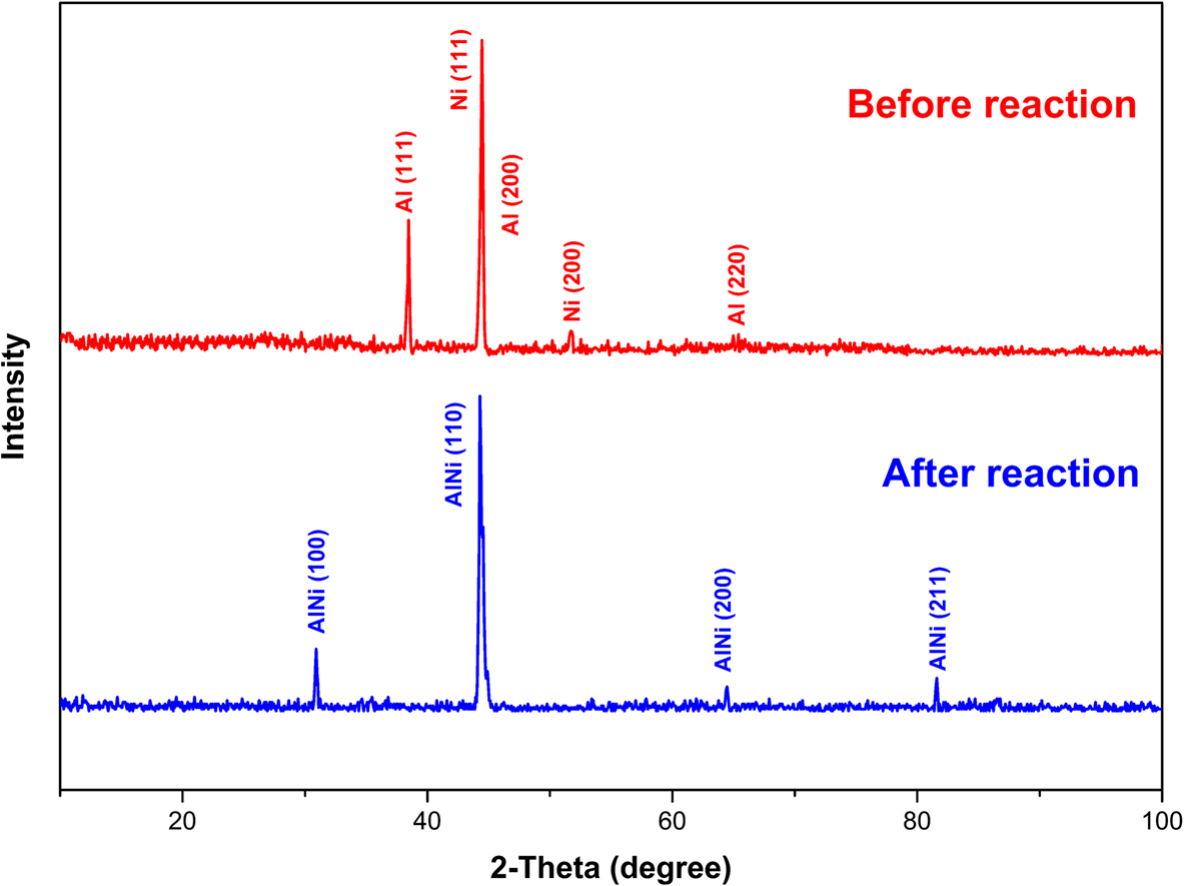
XRD results of the Al/Ni nano-multilayers with bilayer thickness of 200 nm before and after reaction

The thermal properties of Al/Ni nano-multilayers were investigated by DSC, as shown in Fig. 4. Three exothermic peaks can be identified during the heating process. The onset reaction temperature for the first exothermic peak is 503 K, which is less than the melting point of both Al and Ni. The reaction heat of Al/Ni nano-multilayers was calculated through integrating the positive exothermic heat flow which is about 774.6 J/g and almost no mass loss during the test. The low onset reaction temperature and high exothermic heat is conductive to improve the electric explosion process of Cu film bridge. Note that the exothermic heat is well below the maximum theoretical value 1390 J/g [25] and this might be caused by the deviation from the optimum mass ratio and the inevitable surface contamination during the transfer of samples. The phase information of Al/Ni multilayers before and after DSC experiment is detected by XRD, as shown in Fig. 5. Before the reaction, Al and Ni in Al/Ni nano-multilayers are both present in crystalline form. While after the DSC test, all major peaks correspond to AlNi compound, indicating that AlNi is the dominant product of the intermetallic reaction between Al layers and Ni layers.

**Fig. 6:**
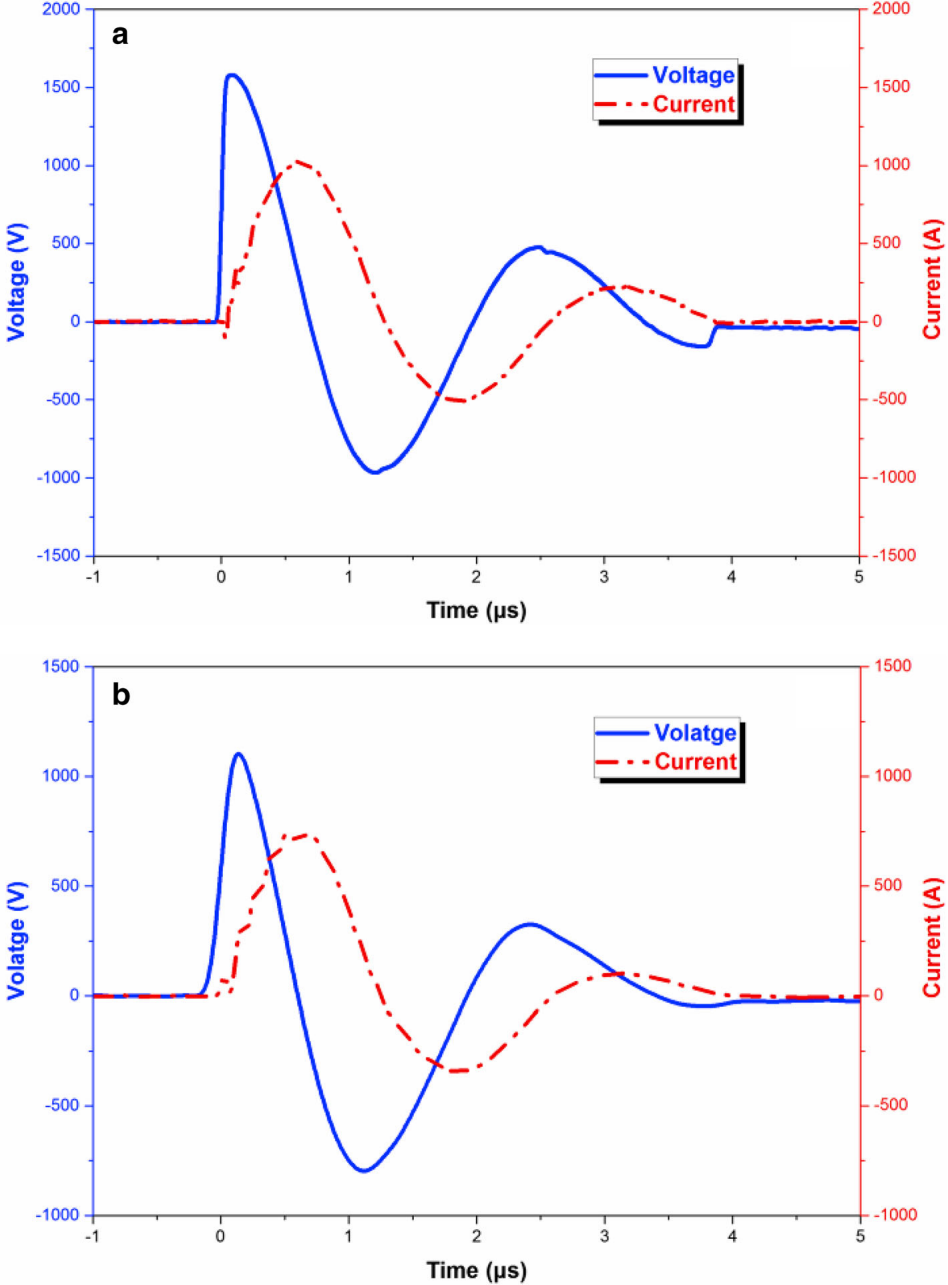
Voltage-current histories for Cu film bridge (**a**) and Cu/Al/Ni-integrated film bridge (**b**) during the explosion processes

When the voltage is applied crossing the bridge area, instantly increasing current density causes the temperature and resistance of bridge to rise rapidly, and the voltage keeps on increasing and reaches the maximal value when the bridge begin to vaporize. Thus, the maximal voltage is defined as the threshold voltage. The typical experimental results of the firing data obtained in the electric explosion tests are shown in Fig. 6. The threshold voltage is measured to be 1580 V for Cu film bridge and 1100 V for Cu/Al/Ni-integrated film bridge. The threshold voltage is reduced around 30% when 2-μm-thick Al/Ni nano-multilayers are integrated with Cu film bridge. The required energy to initiate the Cu film bridge and Cu/Al/Ni-integrated film bridge can be calculated by integrating the product of voltage and current during the electric explosion process. Thus, the initiation energy is about 358.1 mJ for Cu film bridge and about 103.9 mJ for Cu/Al/Ni-integrated film bridge. These results indicate that the energy released by the intermetallic reaction in Al/Ni nano-multilayers can decrease the threshold voltage and initiation energy of Cu film bridge significantly. Specifically, the generated energy of Al/Ni nano-multilayers can be easily tuned by altering the number of layers, which provide a simple method to tailor the electric explosion performances of energetic micro-initiator.

**Fig. 7:**
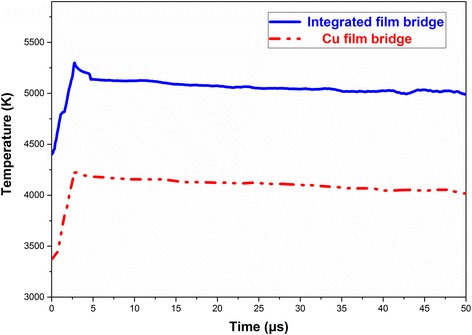
Temperature variation histories for Cu film bridge and Cu/Al/Ni-integrated film bridge during the explosion processes

Figure 7 shows the electric explosion temperature variation of Cu film bridge and Cu/Al/Ni-integrated film bridge under 1800 V discharge voltage. After applying voltage, the explosion temperature reaches the maximum at 4465 K for Cu film bridge and 5300 K for Cu/Al/Ni-integrated film bridge. This increment of the maximum explosion temperature of Cu/Al/Ni-integrated film bridge confirms that the presence of Al/Ni nano-multilayers can increase the total heat energy generated on the Cu film bridge. It is believe that high explosion temperature is beneficial for the expansion of Cu plasma and the consequent improvement of electric explosion performances [14].

**Fig. 8:**
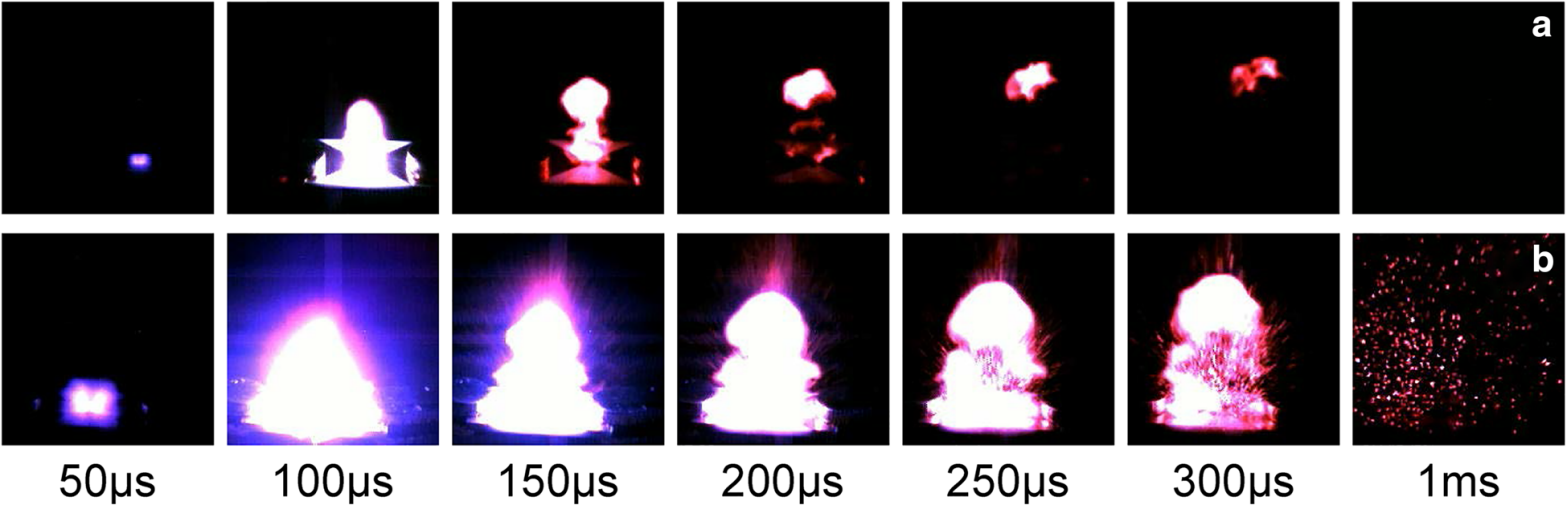
High-speed camera observation of electric explosion processes for Cu film bridge (**a**) and integrated film bridge (**b**)

The electric explosion performances and reaction dynamic processes of Cu film bridge and Cu/Al/Ni-integrated film bridge were recorded simultaneously by high-speed camera, as shown in Fig. 8. The time interval between adjacent pictures is 50 μs, and the specific flame structures in different electric explosion stages have been observed. After triggering, an electric explosion phenomenon accompanied with a bright flash was observed on Cu film bridge, and the duration time of Cu film bridge is about 300 μs. While for Cu/Al/Ni-integrated film bridge, a more fierce combustion process with larger quantities of ejected product particles is observed. The light duration time is over 1 ms, which is much longer than that of Cu film bridge. These results indicate that the intermetallic reaction in Al/Ni nano-multilayers is triggered during the electric explosion process, and the electric explosion performances of Cu film bridge can be improved substantially. The more violent explosion phenomenon of Cu/Al/Ni-integrated film bridge is corresponded well with those results of electric explosion temperature tests.

## Conclusions

In this work, Al/Ni nano-multilayers were integrated with Cu film bridge as nano-energetic material and the electric explosion performances of energetic micro-initiator were investigated. The exothermic reaction in Al/Ni nano-multilayers could be initiated with a quite low onset reaction temperature of 503 K, and the total reaction heat was calculated to be 774.6 J/g. The presence of Al/Ni nano-multilayers on Cu film bridge can improve the maximum of electric explosion temperature as well as decrease the threshold voltage and initiation energy. Compared to Cu film bridge, more fierce combustion process with larger quantities of ejected product particles and longer duration time was observed on Cu/Al/Ni-integrated film bridge. In general, the integration of Al/Ni nano-multilayers with Cu film bridge can improve the electric explosion performances evidently, and the small size of energetic micro-initiator is also beneficial to realize reliable and compact ignition.

## Abbreviations

MEMS: Microelectronic and mechanical systems
SEM: Scanning electron microscopy
TG/DSC: Thermogravimetric analysis/different scanning calorimetry
XRD: X-ray diffraction
